# Editorial: Effects of Physical Exercise on Brain and Cognitive Functioning

**DOI:** 10.3389/fnhum.2022.939112

**Published:** 2022-05-23

**Authors:** Soledad Ballesteros, Laura Piccardi, Joshua Oon Soo Goh

**Affiliations:** ^1^Studies on Aging and Neurodegenerative Diseases Research Group, Department of Basic Psychology II, Universidad Nacional de Educación a Distancia, Madrid, Spain; ^2^Department of Psychology, Sapienza University of Rome, Rome, Italy; ^3^Cognitive and Motor Rehabilitation and Neuroimaging Unit, IRCCS Fondazione Santa Lucia, Rome, Italy; ^4^Graduate Institute of Brain and Mind Sciences, National Taiwan University, Taipei, Taiwan

**Keywords:** active lifestyle, aging, brain plasticity, cognitive functioning, lifespan, physical exercise, wellbeing

This Research Topic (RT) focused on the effects of physical activity (PA) on brain and cognitive functions across the lifespan. A growing body of literature highlights how a physically active lifestyle is associated with reduced risk of dementia, better cognitive functioning, physiological changes in the brain and overall wellbeing. In this vein, studies on people with a sedentary lifestyle highlight an increased risk of cardiovascular diseases and a higher rate of early mortality. However, many studies found contrasting or inconclusive results, depending on different targeted population, tests, and methodologies. Age and activity type seem to influence brain state, but underlying mechanisms remain unclear. This RT addresses how the brain and cognitive functioning is influenced by PA, as well as the psychological benefits of an active lifestyle across the lifespan. It contains 9 contributions on the effect of physical exercise on brain plasticity and cognitive functioning, including original research articles (5), systematic reviews and meta-analyses (3), and a brief research report (1).

How can older adults counteract age-related declines and lead longer, healthier, and fuller working lives? Across the lifespan, the brain exhibits notable plasticity and adapts to environmental changes via modifying brain function and neural connectivity (Goh and Park, 2009; Park and Reuter-Lorenz, 2009; Reuter-Lorenz and Park, 2014). PA, cognitive training, and social engagement are major intervention approaches to improve brain health and cognitive functioning across the lifespan (Hillman et al., 2014; Ballesteros et al., 2015; Donnelly et al., 2016; Kramer and Colcombe, 2018; Ballesteros, 2022). A great deal of research supports that PA protects against age-related cognitive declines enhancing executive functions and memory (Hötting and Röder, 2013; Voelcker-Rehage and Niemann, 2013). This RT aims to provide a current picture of the state of the art on the effect of PA interventions on brain and cognition across the lifespan in healthy individuals and patients with type 2 diabetes mellitus (T2DM).

## Brain Network Modularity of PA in Children, Adolescents, College Students, and Middle-Aged Adults

In their research article, Chaddock-Heymann et al. use brain network modularity as a predictor of training outcomes in 8-to 9-year-old children. In the training but not in the control group, higher modularity of brain networks at baseline was associated with greater improvements in executive functions, cognition, and mathematics. This article is the first to show how the effectiveness of PA on cognition and scholastic performance in children is dependent on baseline brain organization.

The cross-sectional study by Feraco and Meneghetti investigated the relation between years of practicing a sport and both, fluid reasoning and six soft skills. In total 1,115 individuals (10–18 years) filled in a questionnaire for assessing soft skills and completed the Cattel test to assess fluid reasoning. The results confirmed the positive effect of PA on fluid reasoning and adolescents' soft skills.

Salas-Gómez et al. investigated the association between PA with memory and executive functions in a sample of 206 college students. PA was assessed with the IPAQ-SF questionnaire. The authors found that PA correlated with several tests of executive functions, especially inhibitory control. The practice of PA improves the ability to inhibit automatic responses and to show mental flexibility.

Taking a neuroscience perspective, Do et al. used the quantitative approach for coordinate-based activation likelihood estimation (ALE) meta-analysis to find out possible differences in the neural mechanisms underlying motor decision making of experts and novices. A total of 12 studies with 219 motor experts and 210 novices were included in ALE. Greater activation was found for novices compared to experts in the bilateral occipital lobe, left posterior cerebellar lobe, and left middle temporal gyrus. The results suggest that long-term motor training leads to functional reorganization of the brain that is associated with neural efficiency in athletes.

Chou et al. examined the sustained effects of acute resistance training on inhibition in healthy middle-aged adults assigned to exercise or control groups. The resistance exercise consisted of two sets of 7 exercises. The Stroop test was administered before, after training, and 40 min post-training. The findings suggest that a moderate intensity resistance training improves executive functions.

It is well-known that sedentary behavior has negative effects for cognitive performance. Heiland et al. conducted a randomized crossover study to investigate the effects of frequent, short PA breaks, during long sitting periods on cognitive task-related activation of the prefrontal cortex. In the study, 13 middle-aged adults underwent three-3-h seated conditions in which they were interrupted every 30 min by different 3-min break activity (social interaction, walking, or simple resistance activity). Cerebral blood flow decreased in the most difficult memory task after the walking break condition. However, some aspects of working memory performance improved, suggesting a neural efficiency effect. Moreover, mood and alertness improved after walking breaks. Therefore, short walking breaks have positive effect in middle-aged adults to support performance on cognitively demanding tasks.

## Brain and Cognitive Changes Associated With PA and Combined Multi-Domain Interventions in Healthy Older Adults and Patients With T2DM

Polk et al. use a multimodal modeling approach for investigating the effects of aerobic training on regional gray-matter structural integrity in older adults. Results showed that 6-month at-home aerobic exercise 3–4 days a week increased cardiovascular fitness and maintained gray-matter structural integrity in brain areas showing exercise-induced volume changes. Thus, aerobic fitness interventions might contribute to brain maintenance in sedentary older adults.

Rieker et al. conducted a systematic review and multilevel meta-analysis to investigate whether combined training that includes PA and cognitive training would be more effective than single physical or single cognitive training. Fifty studies were included in the meta-analysis involving 6,164 healthy older adults and 783 effects sizes that were submitted to a three-level meta-analysis. The results revealed a small advantage of combined training on cognitive outcomes that was maintained at follow-up. Moreover, combined physical and cognitive training produced some advantage over single cognitive training in improving attention, executive functions, and processing speed. Improvements were highest when the intervention was performed in a social context even though individual training obtained similar results in balance as group training.

The prevalence of T2DM is a global health problem related to unhealthy diet and lack of exercise. T2DM patients have a high risk of developing cognitive decline characterized by long-term explicit memory and executive functions deficits (Redondo et al., 2015, 2016). Ni et al. in their systematic review and meta-analysis investigated whether exercise alone could improve cognition in 738 older patients with T2DM. The results indicated that exercise improved patient global cognition significantly and was not influenced by intervention modality, intervention duration, or cognitive impairment.

In conclusion, the studies included in this RT provide findings on different types of PA interventions as a useful way to change brain and cognitive functioning across the lifespan. Our hope is that this RT will inspire researchers to design and conduct new intervention studies aiming at improving brain health, wellbeing, and cognition at all ages.

## Author Contributions

All authors listed have made a substantial, direct, and intellectual contribution to the work, and approved it for publication.

## Conflict of Interest

The authors declare that the research was conducted in the absence of any commercial or financial relationships that could be construed as a potential conflict of interest.

## Publisher's Note

All claims expressed in this article are solely those of the authors and do not necessarily represent those of their affiliated organizations, or those of the publisher, the editors and the reviewers. Any product that may be evaluated in this article, or claim that may be made by its manufacturer, is not guaranteed or endorsed by the publisher.
